# Editorial: Glycocalyx in Physiology and Vascular Related Diseases

**DOI:** 10.3389/fcell.2021.834280

**Published:** 2022-01-04

**Authors:** Ye Zeng, Bingmei M. Fu

**Affiliations:** ^1^ Institute of Biomedical Engineering, West China School of Basic Medical Sciences and Forensic Medicine, Sichuan University, Chengdu, China; ^2^ Department of Biomedical Engineering, The City College of the City University of New York, New York, NY, United States

**Keywords:** glycocalyx, homeostasis, endothelial cell, vascular related diseases, permeability, mechanotransduction, angiogenesis, inflammation

## Introduction

Glycocalyx (GCX) covers the surface of every mammalian cell. It is closely associated with various physiological and pathophysiological events, including cytoskeleton remodeling, vascular leakage, angiogenesis, and tumor growth and metastasis. Components of GCX are promising biomarkers for many vascular-related diseases, such as sepsis, shock, coronary artery disease, COVID-19, hypertension, diabetes, ischemia-reperfusion injury, and pre-eclampsia. They are also the critical elements involved in the cell-cell and cell-external environment crosstalk in these diseases. The structure of GCX can be modified by bioactive serum constituents, drugs, mechanical forces, and other factors. As an essential signaling mediator, GCX governs the transduction of the extracellular signals into cellular responses. GCX mediated signaling is involved in the cell proliferation, apoptosis, migration, adhesion, autophagy, oxidative stress, angiogenesis, and metastasis.

The mechanisms underlying vascular-related diseases are multifactorial. For example, the interaction between endothelial GCX and the microenvironment may determine the fate of cells and regulate coagulation, permeability, migration, adhesion, and inflammation. Up to date, the research in GCX has focused on elucidating the mechanisms by which the structure and composition of the GCX are disrupted by the pathological factors, with increasing interests in the synthesis and restoration from the disruption. Nevertheless, the composition of GCX is likely to be dynamically altered to maintain the microvascular homeostasis. Enzymatic degradation, *de novo* biosynthesis of new molecules, as well as recruitment of circulating molecules from the blood, all modify the integrity of GCX and its associated functions.

This Research Topic about GCX in physiology and vascular related diseases comprises 15 articles, eight original research and seven review articles, presenting the role of GCX in vascular mechanotransduction, permeability, angiogenesis, as well as the disruption of GCX in various diseases.

### Mechanotransduction

GCX plays a key role in protecting against vascular diseases. Reduced GCX was found in aging vessels and possibly due to stiffness. Mahmoud et al. investigated the stiffness of cell culture substrates on the GCX of various endothelial cells (ECs). They found that heparan sulfate and glypican-1 of GCX in cultured human umbilical vein endothelial cells, rat fat pad endothelial cells, mouse aortic endothelial cell and mouse brain endothelial cells growing on glass were reduced compared to those growing on either soft (stiffness of 2.5 kPa) or stiff (10 kPa) gels. Their study indicates that the glass/stiff substrate could not reflect the true mechanical microenvironment *in vivo*. Numerical methods including macroscopic (continuum-based), mesoscopic [e.g., lattice Boltzmann method (LBM) and dissipative particle dynamics (DPD)] and microscopic [e.g., molecular dynamics (MD) and Monte Carlo (MC) methods] have been used to investigate the complex response of GCX to mechanical stimuli from blood flow. Jiang et al. summarized recent developments in numerical simulation of mechanotransduction of GCX to shear stress, and mechanotransduction initiated biochemical processes such as activation of endothelial nitric oxide synthase (eNOS), and release of Ca^2+^.

### Permeability

The GCX is the primary molecular filter of the blood vessel to macromolecules between blood and surrounding tissues. Direct current stimulation (DCS) has been used to treat a variety of brain disorders, but the mechanism is not clear. Xia et al. demonstrated that DCS increases the blood-brain barrier (BBB) permeability by disrupting the GCX (including heparan sulfate and hyaluronic acid) and tight junction ZO-1 of the BBB and the disruption is NO dependent. The disruption is transient *in vivo* and thus backups the safety of DCS in the clinical application.

It is well established that in the steady state, reabsorption of fluid into microvessels does not occur in tissues such as skeletal muscle due to the balance of hydrostatic and colloid osmotic forces across the GCX. Curry and Michel extended this idea to demonstrate that transient changes in vascular pressure favoring initial reabsorption from the interstitial fluid of skeletal muscle result in much less fluid exchange than is commonly assumed. It is hard to explain the GCX as a filter to macromolecules if the pore size (inter-fiber spacing) is 19.5 nm measured by transmission electron microscopy (TEM). Arkill re-interpretated this pore size as the spacing between proteoglycan core proteins with the collapsed glycosaminoglycan (GAG) side chains during sample preparation in TEM. A new model consisting of the core proteins with surrounding GAGs was proposed to explain the measured reflection coefficient of albumin for the sieving function of EC GCX.

### Angiogenesis, Inflammation, and Vascular Related Diseases

GCX plays a critical role in vessel formation and maturation. Dysfunctional GCX was found in a variety of diseases. Cano et al. revealed that galectin-3 promotes angiogenesis via activation of vascular endothelial growth factor receptor 2 (VEGFR2) in the presence of exogenous VEGF but VEGF-induced VEGFR2 activation is not dependent on galectin-3. The defined pathway of GCX in angiogenesis could aid the anti-VEGF therapies for aberrant angiogenesis and associated vascular permeability and tissue dysfunction. Hu et al. further reviewed the role of GCX in angiogenesis, inflammation, diabetes and COVID-19. Puchwein-schwepcke et al. specifically summarized the role of GCX in physiology and pathology in neonates, infants, and children. Qu et al. reviewed the key role of the GCX in the inflammatory process, highlighted the interaction between inflammatory cytokines release and GCX disruption, and discussed the role of GCX in regulating inflammasomes in inflammation-related diseases such as atherosclerosis and diabetes.

Villalba et al. reviewed the structure and function of GCX across the microvasculature in different organs and the GCX disruption in pathological conditions. New pharmacological interventions attenuating GCX degradation and integrity dysfunction have been summarized. Bush et al. specifically described characteristics of EC GCX breakdown in malaria and discuss how these relate to vascular dysfunction and adverse outcomes. Schenck et al. reviewed the potential role of the GCX damage in the delayed cerebral ischemia (DCI) following an aneurysmal subarachnoid hemorrhage. It was proposed that a damaged GCX could be a potential catalysator of oxidative stress and reduced NO availability in DCI. They also highlighted the potential and limitations of methods currently used to evaluate the glycocalyx, and strategies to restore or prevent glycocalyx shedding. Koch et al. provided evidence of dermal GCX loss (ulex europaeus agglutinin-1) and endothelial dysfunction in patients with chronic kidney disease (CKD). Volume overload rather than inflammation is closely associated with dermal GCX loss, arguing for strict volume control in CKD.

Regarding the GCX in diabetes, Sampei et al. investigated the disruption of GCX in extended inflammation during endotoxemia in a diabetic mouse model. They found a lower expression of GCX synthesis-related genes including *Ext1*, *Csgalnact1*, and *Vcan* in C57BLKS/J Iar^−^ +lepr^db/^lepr^db^ mice than in C57BLKS/J Iar^−^m^+^/+lepr^db^ mice, which is associated with the delayed migration of inflammatory cells in the presence of lipopolysaccharide. Reduced circulating and retinal H_2_S levels are closely associated with microvascular dysfunction in diabetes. Allen et al. found that H_2_S donors prevented high glucose-induced loss of GCX, and increased monocyte adhesion. They also showed that the pretreatment by donors of H_2_S prevented vascular permeability increase in retina in the streptozotocin-induced diabetic rats.

## Concluding Remarks

GCX plays critical roles in EC mechanotransduction, permeability, angiogenesis, and progression of various vascular-related diseases. The reduction in specific components of GCX is closely associated with EC dysfunction and tissue injury, whereas the increased circulating GCX degradation components serve as biomarkers. These findings also suggest that preservation or restoration of GCX is a promising therapeutic strategy for vascular-related diseases. Since the GCX from specific cells is sensitive to mechanical microenvironment, a better substrate should be employed for exploring the endothelial cell biology. Nevertheless, challenges remain in intrinsic complexity, detection technique, *in vitro*/*in vivo* visualization, and drugs or interventions for persevering and restoring GCX. In addition, the details of GCX signaling cascades in the vascular physiology and pathology are yet to be elucidated.

